# Placental Transport of Zidovudine in the Rhesus Monkey

**DOI:** 10.1155/S1064744993000316

**Published:** 1993

**Authors:** Louis E. Ridgway III, Thomas S. King, George I. Henderson, Steven Schenker, Robert S. Schenken

**Affiliations:** 1 Department of Obstetrics and Gynecology, Medicine, and Pharmacology University of Texas Health Science Center at San Antonio San Antonio TX USA; 2 Department of Gynecology and Obstetrics University of Kansas School of Medicine 3901 Rainbow Boulevard Kansas City KS 66160-7316 USA

## Abstract

*Objective:* This study was undertaken to characterize the pharmacokinetics of zidovudine (ZDV) and ZDV-glucuronide (ZDVG) in the material and :fetal circulations of the rhesus monkey.

*Methods:* Cannulas were placed in the maternal external jugular and the fetal internal jugular and carotid artery in 8 pregnant monkeys at .120–130 days gestation. ZDV (3.5 mg/kg) was administered to 5 monkeys and ZDVG (3.5 mg/kg) to 3 monkeys as single intravenous bolus infusions through the maternal catheter. Maternal and fetal blood , samples were collected every 20 min for the first 2 h and then every hour for the next 4 h. Maternal and fetal concentrations of ZDV and ZDVG were determined using high, performance liquid chromatography (HPLC) with ultraviolet (UV) detection.

*Results:* In monkeys who received ZDV, the terminal half-life (T1/2) for ZDV was 37±15 and 33 ± 13 min in the maternal and fetal compartments, respectively. The apparent T1/2 for maternal ZDVG was 124 ± 44 and 142 ± 50 min in the maternal and fetal compartments, respectively. Peak levels of ZDV and ZDVG in the fetal compartment were reached 40 min after injection. The mean fetal/maternal concentration ratios for ZDV and ZDVG ranged from 0.20 ± 0.20 at 20 min to a maximum of 0.74 ± 1.0 at 120 min and from 0.28 ± 0.08 at 20 min to 1.4 ± 1.3 at 180 min, respectively. In monkeys who received ZDVG, the T1/2 for ZDWG in the maternal and fetal compartments was 47 ± 26 and 119 ± 164 min, respectively. ZDVG reached its peak in the fetal compartment at 60 min post-injection. The fetal/maternal rafio ranged from 0.08 ± 0.11 at 20 min to 4.2 ± 4.2 at 180 min post-injection.

*Conclusions: * These data demonstrate that 1) ZDV and ZDVG rapidly cross the placenta to the fetal compartment, 2) ZDV crosses more rapidly than ZDVG, and 3) some metabolism of ZDV to ZDVG occurs in the fetal compartment.


Infectious Diseases in Obstetrics and Gynecology 1:137–143 (1993)

© 1994 Wiley-Liss, Inc.

### Placental Transport of Zidovudine in the Rhesus Monkey

**Louis E. Ridgway III, Thomas S. King, George I. Henderson, Steven Schenker, and Robert S. Schenken**

*Departments of Obstetrics and Gynecology, Medicine, and Pharmacology, University of Texas Health Science Center at San Antonio, San Antonio, TX*

### Abstract

***Objective:*** This study was undertaken to characterize the pharmacokinetics of zidovudine (ZDV) and ZDV-glucuronide (ZDVG) in the material and fetal circulations of the rhesus monkey.

***Methods:*** Cannulas were placed in the maternal external jugular and the fetal internal jugular and carotid artery in 8 pregnant monkeys at 120–130 days gestation. ZDV (3.5 mg/kg) was administered to 5 monkeys and ZDVG (3.5 mg/kg) to 3 monkeys as single intravenous bolus infusions through the maternal catheter. Maternal and fetal blood samples were collected every 20 min for the first 2 h and then every hour for the next 4 h. Maternal and fetal concentrations of ZDV and ZDVG were determined using high-performance liquid chromatography (HPLC) with ultraviolet (UV) detection.

***Results:*** In monkeys who received ZDV, the terminal half-life (T1/2) for ZDV was 37 ± 15 and 33 ± 13 min in the maternal and fetal compartments, respectively. The apparent T1/2 for maternal ZDVG was 124 ± 44 and 142 ± 50 min in the maternal and fetal compartments, respectively. Peak levels of ZDV and ZDVG in the fetal compartment were reached 40 min after injection. The mean fetal/maternal concentration ratios for ZDV and ZDVG ranged from 0.20 ± 0.20 at 20 min to a maximum of 0.74 ± 1.0 at 120 min and from 0.28 ± 0.08 at 20 min to 1.4 ± 1.3 at 180 min, respectively. In monkeys who received ZDVG, the T1/2 for ZDVG in the maternal and fetal compartments was 47 ± 26 and 119 ± 164 min, respectively. ZDVG reached its peak in the fetal compartment at 60 min post-injection. The fetal/maternal ratio ranged from 0.08 ± 0.11 at 20 min to 4.2 ± 4.2 at 180 min post-injection.

***Conclusions:*** These data demonstrate that 1) ZDV and ZDVG rapidly cross the placenta to the fetal compartment, 2) ZDV crosses more rapidly than ZDVG, and 3) some metabolism of ZDV to ZDVG occurs in the fetal compartment. © 1994 Wiley-Liss, Inc.

Key words

Zidovudine, zidovudine-glucuronide, *Macacca mulatta*, pharmacokinetics

In the United States there are an estimated 6,000 pregnancies annually in women who are human immunodeficiency virus (HIV) positive.[1] Treatment with zidovudine (ZDV) is recommended for non-pregnant HIV-infected individuals whose CD4 cell counts drop below 500/mm$^3$.[2] In the pregnant patient, treatment with ZDV is not specifically recommended until the CD4 cell count drops below 200/mm$^3$.[3] Some feel ZDV treatment should be offered during pregnancy when CD4 counts drop below 500/mm$^3$.[3] Regardless of when treatment is initiated, it is apparent that an increasing number of pregnant women and their fetuses are being exposed to ZDV.[4]

While there is considerable information on the pharmacology and metabolism of ZDV in men,

Address correspondence/reprint requests to Dr. Louis E. Ridgway III, Department of Gynecology and Obstetrics, University of Kansas School of Medicine, 3901 Rainbow Boulevard, Kansas City, KS 66160-7316.

Received August 3, 1993
Accepted December 6, 1993

Basic Science Article there are limited data on the pharmacokinetics of ZDV in the pregnant mother and her fetus. Hankins et al.[5] examined maternal, fetal, and amniotic fluid levels of ZDV and ZDV-glucuronide (ZDVG) after multiple oral doses in near-term pregnant baboons. In their study, maternal and fetal levels of ZDV and ZDVG were not significantly different at sampling. However, amniotic fluid levels of ZDV and ZDVG were significantly greater than maternal levels. The authors do not give sampling time after oral dose and no fetal or maternal pharmacokinetic information can be derived from their study. A recent phase I study of ZDV in women in the third trimester of pregnancy[6] demonstrated the pharmacokinetic parameters to be similar to non-pregnant adults. Unfortunately, no information on fetal pharmacokinetics can be obtained in human pregnancies.

In the present study, we utilized a chronically cannulated rhesus monkey model to assess the pharmacokinetics of ZDV and ZDVG in both the maternal and fetal circulations after maternal injection of ZDV or ZDVG.

### SUBJECTS AND METHODS

### Primate Husbandry

Rhesus monkeys (*Macacca mulatta;* n = 8) of 120–130 days gestation (term = 167 days) were studied. Weights ranged from 6.4 to 11.8 kg and all were drug naive. The animals were individually caged in climate-controlled rooms and provided with water ad libitum and food twice daily. The study was approved by the Institutional Animal Care and Use Committee.

### Catheterization

After the monkey was sedated with ketamine hydrochloride (15 mg/kg, i.m.), a cuffed endotracheal tube was placed and anesthesia maintained using a combination of halothane, nitrous oxide, and oxygen. Maternal stability was assured by continuous monitoring of heart rate and blood pressure. The monkey's neck was prepared and draped for surgery. The skin was incised over the right sternocleidomastoid, and the maternal external jugular vein was exposed and cannulated with a polyvinyl catheter, which was advanced to the level of the superior vena cava. The catheter was then tunneled subcutaneously over the right scapula and exteriorized at a point midway between the scapulae.

The fetal position and placental location were determined using real-time ultrasonography. A midline incision was made through the abdominal wall after the maternal abdomen was sterilely prepared and draped. A 3–4 cm transverse uterine incision was made over the fetal head. The chorion and amnion were incised after removal of the amniotic fluid, which was stored at 37°C. The fetal skin overlying the sternocleidomastoid muscle was elevated into the uterine incision. A 2 cm incision was made and a self-retaining retractor placed. The fetal internal jugular vein and carotid artery were exposed and catheterized using polyvinyl catheters. The fetal neck and uterus were closed in layers and the amniotic fluid was replaced. The fetal catheters were then tunneled subcutaneously and exteriorized at the same point as the maternal catheter. The abdomen was closed in layers and the monkey placed in a vest with a mobile tether to allow blood collection without anesthesia.

### Drug Injection and Blood Collection

ZDV (3.5 mg/kg; 3′-azido-3′-deoxythymidine; Sigma Chemical Co., St. Louis, MO) was given as a bolus injection through the maternal catheter in 5 animals after they had fully recovered from generalized anesthesia. ZDVG (3.5 mg/kg; Sigma Chemical Co.) was given to 3 other animals as a bolus injection. Each ZDVG animal received 2 doses of ZDVG 24 h apart. Maternal and fetal samples were collected every 20 min for the first 2 h and then every hour for the next 4 h.

### ZDV and ZDVG Assay

Serum concentrations of ZDV and its major metabolite ZDVG were determined by solid-phase (cartridge $C_{18}$ columns: Bond Elut, Analytichem International, Harbor City, CA) extraction of each serum sample and subsequent analysis by high-performance liquid chromatography (HPLC) with ultraviolet (UV) detection (modified from Good et al.[7]). Briefly, a 50 μl aliquot of each extracted sample was injected onto a Supelcosil LC-18-S column [250 × 4.6 mm; 5 μm particle size (Supelco, Bellefonte, PA)]. The mobile phase consisted of 0.5 M $KH_2PO_4$ (pH 2.7) and $CH_3CN$ (88:12, v/v) at a low rate of 1.0 ml/min. The UV detector was set at a sensitivity of 0.02 AUFS and 254 mm wavelength. Retention times for the internal standard (3′-azido-3′-deoxyuridine, Sigma Chemical

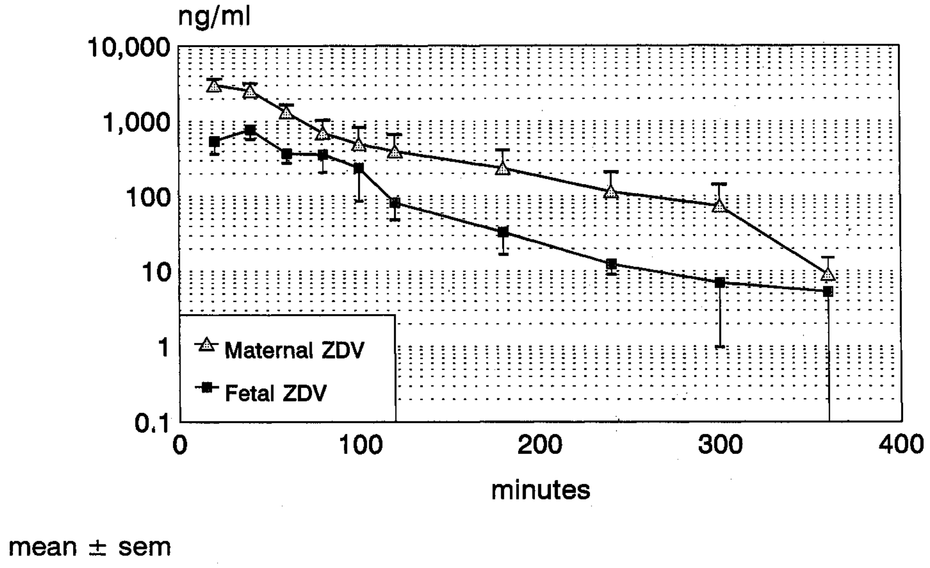

**Fig. 1. Concentrations (mean ± SEM) of ZDV in maternal and fetal circulations following maternal administration of ZDV (3.5 mg/kg) at time 0; n = 5.**

Co.), ZDV, and ZDVG were approximately 4.0, 7.3, and 10.9 min, respectively. Concentrations were determined by comparison of peak areas with those of standards using a programmed integrator interfaced with the UV detector. Detection limits for ZDV and ZDVG were 0.008 and 0.01 μg/ml, respectively. Values were linear through 10 μg/ml of authentic ZDV and ZDVG standards. Intra-assay variation was 5.1% for ZDV and 5.9% for ZDVG.

### Statistics

Pharmacokinetic parameters were estimated using a non-compartmental model and the computer-modeling program MK MODEL. The terminal half-life (T1/2) was estimated using the log-linear model with the least-squares regression of the terminal portion of the plasma concentration curve utilizing the formula T1/2 = 0.693/ke, where ke is the elimination rate constant. Using the iterated data, we estimated the clearance (Cl) and volume of distribution (Vd) by dividing the dose of the drug by the area under the curve and substituting this value into the formula T1/2 = 0.693*Vd/Cl. Wilcoxon sign-rank and rank-sum tests were used where appropriate. $P < 0.05$ was considered to be significant.

### RESULTS

The concentration profiles for ZDV and ZDVG in the maternal and fetal circulations for animals receiving ZDV are shown in Figures 1 and 2. Figure 3 shows the maternal and fetal concentrations of ZDVG in animals who received ZDVG. Table 1 shows the fetal/maternal ratio over the sampling interval for the animals given ZDV and ZDVG. If the serum concentration in the numerator or denominator was zero, the time value was not used in the calculation of the ratios. Tables 2 and 3 summarize the pharmacokinetic parameter estimates for ZDV and ZDVG. In animals receiving ZDVG, complete data were not obtained in monkey 3 for maternal levels after the first dose and the fetal levels after the second dose. Other placental metabolites of AZT have been reported[8,9] and were not sought in this study.

In the animals receiving ZDV, the maternal and fetal T1/2 for ZDVG were significantly longer than for ZDV ($P < 0.016$). Peak fetal levels of ZDV and ZDVG were both reached at 40 min post-injection. In animals receiving ZDVG, peak levels of ZDVG in the fetal compartment were reached at 60 min. The fetal/maternal ZDVG ratio was consistently higher than that of ZDV.

### DISCUSSION

ZDV is being used with increasing frequency in the treatment of pregnant women with acquired immunodeficiency syndrome (AIDS).[3,4] To date there is limited information on the in vivo placental transport of ZDV and its major glucuronidated metabo-

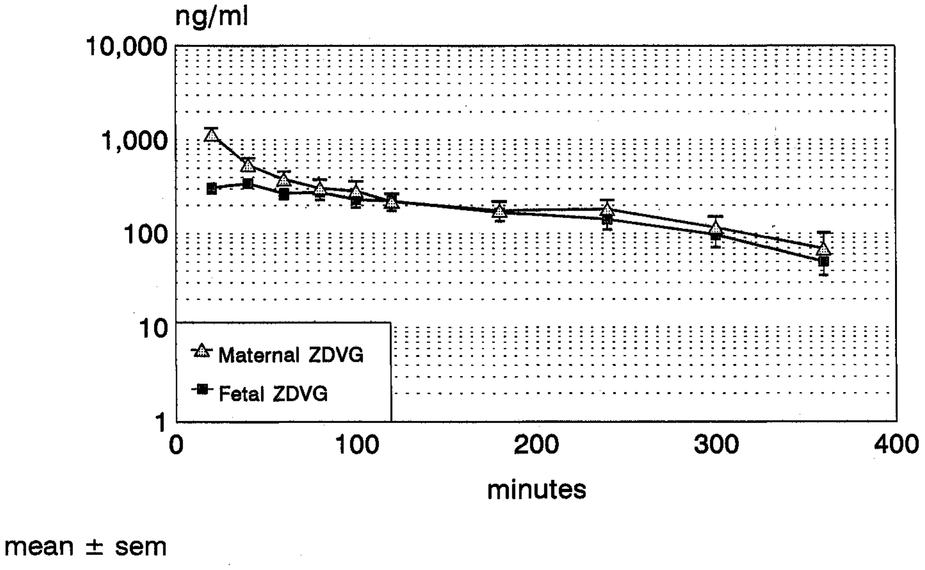

Fig. 2. Concentrations (mean ± SEM) of ZDVG in maternal and fetal circulations following maternal administration of ZDV (3.5 mg/kg) at time 0; n = 5.

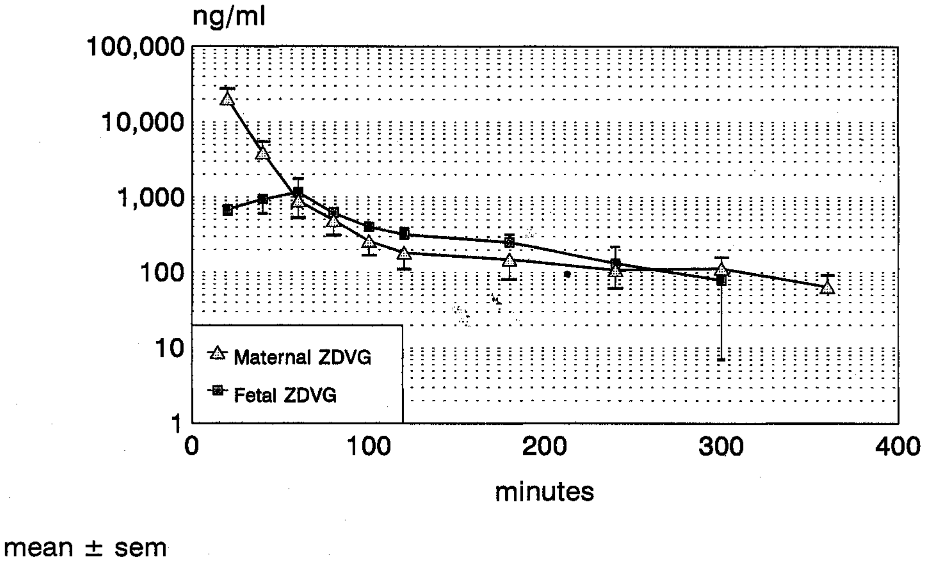

Fig. 3. Concentrations (mean ± SEM) of ZDVG in maternal and fetal circulations following maternal administration of ZDVG (3.5 mg/kg) at time 0; n = 3.

lite ZDVG in pregnancy. The rhesus monkey model, with a placentation very similar to the human, provides a useful approach to the pharmacokinetic study of ZDV and ZDVG in pregnancy. The model provides useful quantitative information as to the elimination of the parent drug and its metabolite in the maternal compartment, as well as its transfer across the placenta and disposition in the fetal circulation. However, it is limited as to mechanistic data on the nature of the placental transfer process. The latter is best investigated using the isolated perfused placental cotyledon system, as employed by us[8] and others[9,10] earlier.

Using the in vivo model, we show clearly that 1) ZDV is removed rapidly from the maternal circulation and appears quickly in the fetal plasma, 2)

**TABLE 1. Fetal/maternal ratio**

| Time | Monkeys given ZDV | | Monkeys given ZDVG |
|---|---|---|---|
| (min) | ZDV (n) | ZDVG (n) | ZDVG (n) |
| 20 | 0.20 ± 0.21 (5) | 0.28 ± 0.08 (5) | 0.08 ± 0.11 (4) |
| 40 | 0.40 ± 0.33 (5) | 0.67 ± 0.17 (5) | 0.52 ± 0.61 (4) |
| 60 | 0.35 ± 0.21 (5) | 0.81 ± 0.31 (5) | 2.17 ± 0.99 (4) |
| 80 | 0.68 ± 0.46 (5) | 1.19 ± 0.76 (5) | 2.65 ± 1.7 (4) |
| 100 | 0.65 ± 0.69 (5) | 1.12 ± 0.92 (5) | 2.47 ± 0.93 (4) |
| 120 | 0.74 ± 1.0 (5) | 1.19 ± 0.70 (5) | 2.72 ± 0.71 (4) |
| 180 | 0.48 ± 0.68 (5) | 1.43 ± 1.3 (5) | 4.19 ± 4.2 (3) |
| 240 | 0.45 ± 0.50 (3) | 1.42 ± 1.7 (5) | 2.32 ± 1.9 (3) |
| 300 | 0.86 ± — (1) | 0.70 ± 0.32 (4) | 1.45 ± 0.64 (2) |
| 360 | 0.92 ± — (1) | 0.76 ± 0.73 (3) | — |

ZDVG is generated rapidly as a result of ZDV metabolism in the maternal compartment, and 3) ZDVG crosses the placenta into the fetal circulation, albeit apparently more slowly than ZDV.

The T1/2 of ZDV in the maternal circulation is comparable to values reported earlier in non-pregnant[11] and pregnant women,[6,12] as well as in the pregnant monkey model.[13] Moreover, the clearance and volume of distribution of ZDV in our studies approximate the data of Lopez-Anaya et al.[13] in the pregnant monkey and O'Sullivan et al.[6] in pregnant women. The plasma binding of ZDV is low;[13] hence, the low albumin concentration seen in pregnancy should have no effect on ZDV clearance. It appears, therefore, that the pharmacokinetics of ZDV in pregnancy are similar to those seen in non-pregnant patients. Moreover, in our model, there was rapid formation of ZDVG from injected ZDV, with peak maternal ZDVG already evident on initial sampling. The T1/2 of ZDVG in the maternal circulation after ZDV administration was much longer than the T1/2 of ZDV. This may be explained by ZDVG derivation from ZDV. This interpretation is consistent with lower ZDVG than ZDV concentrations in maternal plasma and by a shorter ZDVG T1/2 in maternal plasma when the agent is administered as ZDVG. In other studies, a rapid formation of ZDVG from ZDV was also noted after ZDV administration, although the half-life of both drugs was very similar.[13] Similarly, in our studies, clearances of administered ZDV and ZDVG in the maternal circulation were almost identical.

Both ZDV and ZDVG appeared rapidly in the fetal circulation after ZDV administration. The T1/2 of ZDV in the fetal compartment was not significantly different from that in maternal circulation. In order to determine whether the ZDVG present in the fetal circulation is a result of placental transfer or fetal metabolism, we looked at the monkeys given ZDVG alone. After ZDVG administration to the mother, peak ZDVG in the fetal circulation was noted at 60 min. ZDVG disappeared more slowly than ZDV from the fetal circulation and the fetal/maternal ZDVG ratio was consistently greater than the ZDV ratio. The fetal/maternal ZDVG ratio after maternal ZDVG administration was also lower at 20 and 40 min than after ZDV administration, but this was substantially reversed thereafter. The most likely explanations for these composite findings are that the transfer of ZDVG from maternal to fetal circulations is slower than ZDV passage across the placenta and some metabolism of ZDV to ZDVG occurs in the fetal compartment. This is notwithstanding the known relative immaturity of fetal hepatic enzymes from glucuronidation.[14–16] Moreover, the rapid clearance of ZDVG from the maternal circulation and the slower transfer of ZDVG across the placenta probably contribute to the retention of this metabolite in the fetus and the increased fetal/maternal ratio. Other investigators[5] have reported high levels of ZDVG in fetal serum and amniotic fluid of pregnant monkeys given multiple oral doses of ZDV and have proposed that the amniotic fluid may serve as a reservoir for ZDVG. Verification of this overall concept will require infusions of both ZDV and ZDVG into the *fetal* compartment with monitoring of relative rates of transfer of each substance into the maternal circulation and into fetal bile and urine (amniotic fluid). Preliminary data in our unit, however, using the perfused isolated placental cotyledon support a more rapid diffusion of ZDV than of ZDVG across the human placenta. This is consistent with the greater polarity of ZDVG[8,9] and with the concept that these drugs cross the placenta passively.[8–10]

While transfer of animal data to clinical medicine is difficult, our data suggest that ZDV dosing does not need to be modified in pregnancy as ZDV should not accumulate in the fetal compartment. While ZDVG may accumulate in this compartment, this should not be clinically important as long as the metabolite remains inactive.

In conclusion, our data along with those of the previously cited investigators suggest that both ZDV

**TABLE 2. Pharmacokinetic parameter estimates in monkeys given ZDV**

| | T½ (min) | | | | Maternal ZDV | |
|---|---|---|---|---|---|---|
| Monkey No. | Maternal ZDV | Fetal ZDV | Maternal ZDVG | Fetal ZDVG | Vd (ml/kg) | Cl (ml/min/kg) |
| 1 | 45 | 28 | 148 | 229 | 667 | 12 |
| 2 | 56 | 54 | 169 | 138 | 806 | 8 |
| 3 | 22 | 37 | 52 | 120 | 379 | 13 |
| 4 | 23 | 25 | 125 | 103 | 487 | 16 |
| 5 | 37 | 20 | 123 | 120 | 981 | 39 |
| Mean | 37 | 33 | 124 | 142 | 664 | 18 |
| SD | 15 | 13 | 44 | 50 | 242 | 12 |

**TABLE 3. Pharmacokinetic parameter estimates in monkeys given ZDVG**

| | T½ (min) | | Maternal ZDVG | |
|---|---|---|---|---|
| Monkey No. | Maternal ZDVG | Fetal ZDVG | Vd (ml/kg) | Cl (ml/min/kg) |
| 1 | 42 | 51 | 1,931 | 39 |
| | 37 | 42 | 428 | 10 |
| 2 | 12 | 36 | 26 | 20 |
| | 70 | 413 | 840 | 13 |
| 3 | — | 54 | — | — |
| | 74 | — | 1,417 | 13 |
| Mean | 47 | 119 | 928 | 19 |
| SD | 26 | 164 | 761 | 12 |

and ZDVG rapidly appear in the fetal circulation following maternal ZDV injection. Placental transfer most likely occurs by passive diffusion with ZDV crossing more rapidly than ZDVG crosses. ZDVG may then accumulate in the fetal compartment with amniotic fluid serving as a reservoir. Fetal ZDVG accumulation appears to result from diffusion of maternal ZDVG and from fetal metabolism of ZDV accompanied by slow back diffusion of fetal ZDVG and its decreased excretion by fetal organs.

### ACKNOWLEDGMENTS

We gratefully acknowledge the technical support of Arturo Moreno and Diane Frasier and the assistance of Gretta Small and Patricia Littleton in the preparation of the manuscript. This work was supported by NIH Career Development (RSDA) KO 2 AA00 121 and by NIAAA R01 HD 26707.

### REFERENCES

1. Centers for Disease Control: HIV prevalence estimates and AIDS case projections for the United States: Report based upon a workshop. Morbidity and Mortality Weekly Report 39(RR-16):1–31, 1990.
2. Volberding PA, Lagakos SW, Koch MA, et al.: Zidovudine in asymptomatic human immunodeficiency virus infection: A controlled trial in persons with fewer than 500 CD4 positive cells per cubic millimeter. N Engl J Med 322:941–949, 1990.
3. HIV Infections. ACOG Technical Bulletin. Number 169, June 1992. The American College of Obstetricians and Gynecologists, Washington, DC.
4. Minkoff HL, Moreno JD: Drug prophylaxis for human immunodeficiency virus-infected pregnant women: Ethical considerations. Am J Obstet Gynecol 163:1111–1114, 1990.
5. Hankins ZDV, Lowery CL, Scott RT, et al.: Transplacental transfer of zidovudine in the near-term pregnant baboon. Am J Obstet Gynecol 163:728–732, 1990.
6. O'Sullivan MJ, Boyer PJ, Scott GB, et al.: The pharmacokinetics and safety of zidovudine in the third trimester of pregnancy for women infected with human immunodeficiency virus and their infants: Phase I Acquired Immunodeficiency Syndrome Clinical Trials Group study (protocol 082). Am J Obstet Gynecol 168:1510–1516, 1993.
7. Good SS, Reynolds DJ, de Miranda P: Simultaneous quantification of zidovudine and its glucuronide in serum by high performance liquid chromatography. J Chromatogr 431:123–133, 1988.
8. Schenker S, Johnson RF, King TS, Schenken RS, Henderson GI: Azidothymidine (zidovudine) transport by the human placenta. Am J Med Sci 299(1):16–20, 1990.
9. Liebes L, Mendoza S, Wilson D, Dancis J: Transfer of zidovudine (AZT) by human placenta. J Infect Dis 161:203–207, 1990.
10. Bawdon RE, Sobhi S, Dax J: The transfer of anti-human immunodeficiency virus nucleoside compounds by the term human placenta. Am J Obstet Gynecol 167:1570–1574, 1992.
11. Klecker RW Jr, Collins JM, Yarchoan R, et al.: Plasma and cerebrospinal fluid pharmacokinetics of 3′-azido-3′-deoxythymidine: A novel pyrimidine analog with potential application for the treatment of patients with AIDS and related diseases. Clin Pharmacol Ther 41:407–412, 1987.
12. Watts DH, Brown ZA, Tartaglione T, et al.: Pharmacokinetic disposition of zidovudine during pregnancy. J Infect Dis 163:226–232, 1991.

13. Lopez-Anaya A, Unadkat JD, Schumann LA, Smith AL: Pharmacokinetics of zidovudine (azidothymidine). III. Effect of pregnancy. J Acquired Immune Deficiency Synd 4:64–68, 1991.
14. Dutton GJ: Developmental aspects of drug conjugation with special reference to glucuronidation. Annu Rev Pharmacol Toxicol 18:17–35, 1978.
15. Schenker S, Dawber NH, Schmid R: Bilirubin metabolism in the fetus. J Clin Invest 43:32, 1964.
16. Bashore RA, Smith F, Schenker S: Placental transfer and disposition of bilirubin in the pregnant monkey. Am J Obstet Gynecol 103:950, 1969.
